# Efficiency evaluation of Chinese Yunnan Province County Area Public Service for sports and fitness based on three-stage DEA model

**DOI:** 10.1371/journal.pone.0340803

**Published:** 2026-02-02

**Authors:** Ziteng Li, Xuguang Wang

**Affiliations:** Sports Humanities and Social Sciences Research Center, Tianjin University of Sport, Tianjin, China; USTC: University of Science and Technology of China, CHINA

## Abstract

County areas hold critical strategic significance in terms of population, gross domestic product, and their role within the National Fitness Strategy and National Fitness Public Service System.Analyzing the efficiency of county areas National Fitness public services in Yunnan Province can provide evidence-based support for advancing the development of a higher-level public service system for national fitness across Southwest China. Using a three-stage Data envelopment analysis (DEA) method, this study evaluates the efficiency of public fitness services at the county-level administrative division in Yunnan Province for the year 2023. The analysis encompasses 129 county-level administrative divisions, comprising 17 municipal districts and 112 county areas within Yunnan Province. Research indicates that environmental factors such as regional GDP, sports and fitness infrastructure, local industrial structure, and population density significantly influence the efficiency of national fitness services. After excluding these variables in the second-stage stochastic frontier analysis (SFA) regression analysis, the Technical Efficiency (TE) of national fitness services in Yunnan Province’s municipal districts and county areas reached 0.919 and 0.824 respectively in 2023. Adjusted Scale Efficiency (SE) for municipal districts and county area generally exceeded Pure Technical Efficiency (PTE), with significant PTE disparities between the municipal districts and county area. Additionally, 47.06% of municipal districts and 72.32% of county area exhibited Increasing Returns to Scale (IRS). Accordingly, efforts should be made to continuously improve the county area supervision management system and investment mechanisms, broaden service types to achieve integrated development, and fully leverage IRS to promote the dissemination of regional and ethnic traditional cultures.

## Introduction

The public service system for sports and fitness is an important component of the overall public service system, continuously building a higher level of public service system for fitness and physical health is a vital project for improving people’s well-being and meeting their aspirations for a better life [1]. Since October 2021 to May 2023, China has issued multiple policy documents, including the Guiding Opinions on Promoting the Construction of Sports Parks, the 14th Five-Year Public Service Plan, the Opinions on Building a Higher-Level National Fitness Public Service System, and the Action Plan for the Improvement of National Fitness Venues and Facilities (2023–2025). These policies advocate for promoting the allocation of public service resources toward grassroots county-level areas, grounded in the principles of equity, population as a critical determinant, and counties as the primary implementers.

Within China’s administrative division system, county-level administrative divisions constitute the third tier of administrative units, positioned between prefecture-level divisions and township-level divisions. These divisions encompass counties, autonomous counties, county-level cities, and municipal districts [2]. Among these county-level units, the county represents China’s most traditional and stable form of grassroots governance. It serves as the fundamental structural unit of county-level administrative divisions, possessing a complete system of county-level people’s governments and occupying a significant position within the national grassroots governance framework [3–4]. In contrast, while municipal districts also fall under county-level administrative divisions, they function as administrative units under the jurisdiction of prefecture-level cities. Their administrative structure differs from that of counties, as they do not establish full-fledged county-level governments. Instead, they are directly administered by prefecture-level municipal governments, reflecting distinct management system logics [5]. It should be noted that county area is not an official administrative designation but rather an analytical concept within regional development and governance studies. It emphasizes regional integrity and the significance of integrated urban-rural development. Its scope typically encompasses the overall spatial boundaries of county-level administrative divisions (including counties, autonomous counties, and county-level cities), but excludes municipal districts [6]. Table 1 reveals the structural relationships among county-level administrative divisions, county area, counties and municipal districts.

**Table 1:** Structural relationships among county-level administrative divisions, county area, counties and municipal districts.

Term	Conceptual nature	Administrative level	Considered part of the county area	Authoritative sources
County-level administrative division	Third-tier administrative division category	Encompasses all county-level units	No	National Bureau of Statistics of China; Ministry of Civil Affairs
County area	Statistical and governance research concept	Not an administrative level	–	National Bureau of Statistics of China
County	A type of county-level administrative division	County level	Yes	Ministry of Civil Affairs
Municipal district	A type of county-level administrative division	County level	No	Ministry of Civil Affairs

Participation serves as a pivotal variable linking efficiency, equity, and public health outcomes. Enhancing the efficiency of public sports services—by improving supply quality and accessibility—directly promotes increased sports participation among residents, this mechanism has been validated in public service efficiency studies across multiple countries [7–8]. Meanwhile, the resource conservation and refined governance enabled by efficiency gains facilitate the allocation of more resources to disadvantaged regions or underrepresented groups, thereby enhancing the fairness of sports resource distribution [9–11]. Sports participation, as a direct behavioral pathway influencing health status, can generate significant health benefits across physiological, psychological, and social dimensions through physical activity [12–13]. The equity of public sports services constitutes a crucial structural safeguard for public health outcomes by improving accessibility and usage conditions for disadvantaged groups [14–15]. Participation and equity jointly contribute to the overall improvement of population health levels, the outcomes are reflected not only in reduced risks of chronic diseases and enhanced quality of life, but also in narrowing health disparities [16–17]. Improvements in public health outcomes can in turn enhance public recognition of public investment in sports, thereby strengthening the government’s motivation to increase the supply of sports services and achieve continuous institutional optimization [15, 18]. From the perspective of health determinants in society, how macro-environmental variables such as regional economic development levels, urbanization rates, industrial structures, and population density reshape resource allocation redundancy and economies of scale structures, thereby influencing the transmission chain linking efficiency-participation-equity-public health outcomes—remains to be systematically examined at the county level [19–20]. Within this context, this study constructs an analytical framework centered on the efficiency-participation-equity-public health outcomes continuum to reveal the mechanisms shaping the efficiency of county-level public service for national fitness and their institutional context.

National fitness services and facilities in county areas exhibit significant developmental imbalances across multiple dimensions—including venues, funding, equipment, and personnel—due to disparities and delays in resource allocation [21]. Although gaps in public service for national fitness levels between regions and between urban and rural areas are narrowing, structural imbalances in the supply of public fitness services persist at the county level, this imbalance leads to widespread issues such as low utilization rates and insufficient participation in physical activities [22–24]. In the implementation of public services for national fitness, inadequate evaluation mechanisms for efficiency or performance are prevalent in county-level areas and grassroots regions, assessment outcomes often suffer from superficiality, lack of quantification, and impracticality. There is an urgent need to advance the development of efficiency evaluation indicators for county-level public services for national fitness [24–27].

Current scholarly research on the development level, performance and efficiency of the national fitness public services primarily employs the Delphi method, Analytic Hierarchy Process, Data Envelopment Analysis (DEA)-Tobit, DEA-SBM, spatial difference-in-differences model, Theil indices, importance-performance analysis, and satisfaction three-factor satisfaction analysis to evaluate public sports services by examining multi-dimensional factors such as sports parks, the area/quantity/configuration of sports facilities, the number of social sports instructors, and public sports expenditures, thereby assessing efficiency or performance from the perspectives of human, financial, and facility resources [25–33]. The existing research mainly focuses on macro perspectives such as the country, urban agglomerations, and provincial regions. Few scholars have conducted in-depth explorations on the efficiency of public services for national fitness from micro perspectives such as counties and communities. Moreover, due to the limitations of research methods, the impact of environmental factors on output is often overlooked or difficult to reflect, such as economic level, urbanization rate, industrial structure. Based on this, this study employs a three-stage DEA model and, using Yunnan Province as a case study, examines the efficiency of public services for national fitness at the county-level administrative division through multidimensional factors such as sports facilities, social sports instructors, and public sports expenditures, investigates the impact of environmental factors on the accuracy of efficiency evaluations, and proposes strategies to enhance the efficiency of public services for national fitness in Southwest China based on empirical analysis results.

## Methods

### Sample selection and data sources

Yunnan Province is situated at the intersection of China, Southeast Asia, and South Asia. It is the province in China with the greatest diversity of ethnic groups. Yunnan Province consists of 16 prefectures. Among them, eight cities including Kunming, Zhaotong, Qujing, Yuxi, Baoshan, Pu’er, Lijiang and Lincang have a total of 17 urban districts. The remaining areas of these eight cities including Chuxiong, Honghe, Wenshan, Xishuangbanna, Dali, Dehong, Nujiang and Diqing have a total of 112 counties [34]. Yunnan Province is China’s most ethnically diverse province, featuring a rich variety of county-level administrative divisions that encompass the vast majority of China’s county types, and it is home to 25 ethnic minority counties and 29 ethnic autonomous counties, occupies a unique geographical position as the center of Asia, and includes 25 border counties [35–36]. This study examines 129 county-level administrative regions in Yunnan Province, comprising 17 urban districts and 112 county-level areas, as objects of empirical research. The data regarding indicators such as population, economy, infrastructure, and fiscal investment of these 129 county-level administrative regions are sourced from the China Sports Statistics Yearbook, Yunnan Statistical Yearbook, China Statistical Yearbook (Township), and the statistical yearbooks of each respective county (city).

### Variable selection

Utilizing the concepts of purposefulness,scientific rigor and quantifiability, selected the input indicators, output indicators and environmental variables for evaluating the efficiency of county-level public services for national fitness employing the three-stage DEA model.

Input indicators. In management science, resources in human production activities are categorized as material, human, and financial [37]. Therefore, the input indicators in this study are categorized into three dimensions: venue and facility input, human resource input, and financial input. Considering the current state of public services for national fitness, the allocation of venues and facilities is determined by the per capita sports venue area and the comprehensive venue and facility index. The comprehensive venue and facility index is generated based on the quantity of venues and facilities, including public stadiums, public gymnasiums, sports parks, fitness centers for all people, athletics fields, social football fields, swimming (diving) pools, encompassed within each county-level administrative region and its service radius, with weights assigned.

Environmental variables should reflect the objective constraints faced by each county in terms of economic and social development, population concentration, and industrial structure, rather than inputs that can be directly adjusted by sports authorities in the short term. Based on this, per capita regional GDP, population density, urbanization rate, and the proportion of tertiary industry value added were selected to characterize the external environment embedded in the supply of public services for national fitness. Existing research generally incorporates regional economic development levels and population conditions as environmental variables in the second-stage stochastic frontier analysis when evaluating the efficiency of provincial public sports services, thereby indicating that failing to control for these exogenous factors can systematically overestimate Scale Efficiency (SE) and underestimate Pure Technical Efficiency (PTE) [38–40]. Per capita regional GDP comprehensively reflects a county’s fiscal capacity, residents’ disposable income, and overall public service provision capabilities, and it serves as a core indicator for measuring macro-level development, as confirmed by provincial-level studies on public sports service efficiency [38–39]. Population density reflects the spatial concentration of service recipients, directly influencing the accessibility, utilization rates, and economies of scale of public sports facilities, treating it as an external environmental variable allows for controlling structural differences across counties in terms of service coverage and effective demand scale [41]. Urbanization rates reflect residents’ built environments from both spatial structure and lifestyle dimensions, making them suitable as macro-level contextual indicators influencing public sports service accessibility and organizational efficiency, and not be conflated with sector-controlled inputs [40–42]. The proportion of tertiary industry value added in regional GDP reflects the degree of service-oriented and modernized industrial structure within a county, with its development level directly influencing the vitality of social sports organizations, sports clubs, and the fitness industry [41–42]. Given the significant positive correlation between the sports industry and public health, incorporating the tertiary industry share as an environmental variable helps control for systemic differences in sports-related service ecosystems across different counties.

Output indicators should be able to summarize the effectiveness of public fitness services at both the service delivery and health outcomes levels. The “proportion of residents regularly participating in physical exercise” is widely used in national fitness statistics and empirical research as a core indicator of regional sports participation and public service performance [38–40], which is consistent with the World Health Organization’s 2020 guidelines emphasizing that achieving recommended physical activity levels can significantly reduce all-cause mortality and the risk of cardiovascular and metabolic diseases [43–44]. The physical fitness attainment rate has been widely used in multiple rounds of national physical fitness monitoring to assess regional disparities in residents’ physical fitness and health [45]. Therefore, treating the proportion of the population regularly participating in physical exercise as a behavioral output reflecting service utilization, while viewing the physical fitness attainment rate as an accumulated health outcome output, facilitates a more accurate portrayal of the efficiency of county-level public fitness services within the three-stage DEA framework. The inputs, outputs, environmental variables, and their conceptual roles in county-level public service for national fitness are shown in Table 2.

**Table 2:** The conceptual roles in county-level public service for national fitness of inputs, outputs, environmental variables.

Indicator category	Indicator (operational definition)	Conceptual role in the production of National Fitness public services
Inputs	Fiscal Investment (Per Capita Local General Public Budget Expenditure) category	The scale of financial resources available to local governments for sports and related public services constitutes a fundamental investment element for constructing sports venues and facilities, purchasing equipment, and organizing mass fitness activities.
	Human resources input (number of social sports instructors per 1,000 people)	The human resource density of county-level social sports instructors reflects the level of human capital available to provide residents with scientific fitness guidance, organize activities, and disseminate health knowledge.
	Facility input 1 (per-capita sports venue area)	Reflects the amount and scale of usable sport space per resident; a basic indicator of county-level sport infrastructure.
	Facility input 2 (composite facility index)	The comprehensive assessment of public sports facilities, including public stadiums, gymnasiums, sports parks, fitness centers, community soccer fields, and swimming pools, evaluates their quantity, service radius, and spatial layout. This analysis reflects the rationality of facility types and spatial arrangements, providing a holistic portrayal of their quantity, diversity, and accessibility.
Outputs	Residents’ sport participation (proportion of residents who participate in regular physical exercise)	Directly reflects the level of habitual exercise behavior; a pivotal indicator linking efficiency, equity, and population health outcomes.
	Residents’ physical fitness (pass rate of the National Physical Fitness Measurement Standards among urban and rural residents)	Reflects the combined outcomes of public sport services and residents’ participation in terms of physical function, body composition, cardio respiratory endurance; the final health output in the public service production process.
Environmental variables	Economic development (per-capita GDP)	Proxies overall county-level economic development and residents’ income, shaping local fiscal capacity, infrastructure investment ability, and residents’ sport consumption and leisure demand.
	Sports and Fitness Environment (Regional Urbanization Rate)	Reflecting the concentration of population and built environment, it serves as a significant external environmental factor influencing the layout of sports facilities, public transportation conditions, and the form of recreational spaces.
	Industrial structure (tertiary industry value added/ GDP)	The proportion of the tertiary sector serves as a measure of a region’s transition from traditional industry to a service-based economy. A higher proportion indicates greater potential for the integrated development of public sports services with sports tourism, health industries, and related sectors, thereby influencing the efficiency of public sports service delivery.
	Accessibility constraint of National Fitness public services (population density)	The average spatial distance between residents per unit area and public sports facilities, along with service pressure, constitutes an environmental constraint on the accessibility and affordability of public sports services.

### Model construction

DEA widely utilized efficiency evaluation methodology, can accommodate various input and output indications while avoiding the intricate challenge of establishing indicator weights [46]. When evaluating the efficiency of public services for national fitness, a multitude of input and output indicators are taken into account. These include human resource input, financial investment, the input of sports facilities, and Residents’ sport participation and physical fitness. The DEA model serves as a tool for assessing the effectiveness of various research entities, enabling evaluation from the perspective most conducive to the decision-making unit (DMU). Consequently, this research selects the DEA model to measure the efficiency of public services for national fitness in each county – level administrative division. Specifically, each county-level administrative division is designated as a DMU, and DEA utilized to construct a production frontier. By juxtaposing the input-output characteristics of each county – level administrative division with this frontier, an precise measurement of the efficiency of public services for national fitness at the county level can be achieved, where a greater deviation from the production frontier signifies a lower efficiency value of and a smaller deviation denotes a higher efficiency [47]. Incorporating environmental and random factors into the DEA model places DMUs in identical environmental conditions, enabling more effective calculation of their relative efficiency [48]. This study employs a three-stage DEA model to measure the efficiency of public services for national fitness in each county-level administrative division of Yunnan Province, and conducts quantitative comparative analysis of Technical Efficiency (TE), PTE, and SE of municipal districts and county-level areas.

### Three-Stage DEA model calculation process

The calculation process of the three-stage DEA model encompasses two assumptions: The Charnes-Cooper-Rhodes Model (CCR) and the Banker-Charnes-Cooper Model (BCC) measure efficiency under the assumptions of constant returns to scale (CRS) and variable returns to scale (VRS) respectively [47]. In the first stage, the standard DEA model is employed to assess the effectiveness of Public Services for National Fitness provision across county area and districts, while also deriving input slack variables in terms of human resources, fiscal investment, and facilities. The BCC model incorporates input-oriented and output-oriented calculations, respectively designed to optimize inputs for a given output level and maximize output for a fixed input level. The 129 county-level administrative division in Yunnan Province exhibit significant disparities in population size and economic development. By employing the BCC model, TE can be decomposed into PTE and SE, thereby distinguishing inefficiencies stemming from pure management failures from those arising from scale disadvantages. Accordingly, this study adopts an input-oriented BCC model under the VRS assumption to estimate efficiency scores in the first-stage analysis.

In estimating the efficiency of county-level administrative division public services for National Fitness in this study, the CCR model yields the comprehensive technical efficiency (CRS technical efficiency; crste), whereas the BCC model yields pure technical efficiency (VRS technical efficiency; vrste). Scale efficiency is then obtained as crste/vrste, these measures respectively capture production efficiency attributable to managerial/technical performance and that attributable to scale effects [47]. In the DEA calculation, there are 129 DMUs (representing county-level administrative unit), the input and output of the i DMU are represented by vectors x_i_ and y_i_ respectively. The CCR model with non-Archimedean infinitesimal ε, Eq (1)is as follows:


$$s.t.\left\{ \begin{array}{c} min\left[\theta -\varepsilon \left({\hat{e}}^{T}s^{-}+e^{T}s^{+}\right)\right]=V_{D}(\varepsilon )\\ \sum_{i=\text{1}}^{n}x_{i}\lambda_{i}+s^{-}=\theta x_{i\text{0}}\\ \sum_{i=\text{1}}^{n}y_{i}\lambda_{i}-s^{+}=y_{i\text{0}}\\ \lambda_{i}\geq \text{0},i=\text{1},\text{2},\ldots ,n\\ s^{-}\geq \text{0},s^{+}\geq \text{0} \end{array}\right.\;$$ (1)

The BCC model with non-Archimedean infinitesimal ε, Eq (2)is as follows:


$$s.t.\left\{ \begin{array}{c} min\left[\theta -\varepsilon \left({\hat{e}}^{T}s^{-}+e^{T}s^{+}\right)\right]=V_{D}(\varepsilon )\\ \sum_{i=\text{1}}^{n}x_{i}\lambda_{i}+s^{-}=\theta x_{i\text{0}}\\ \sum_{i=\text{1}}^{n}y_{i}\lambda_{i}-s^{+}=y_{i\text{0}}\\ \sum_{i=i}^{n}\lambda_{i}=\text{1}\\ \lambda_{i}\geq \text{0},i=\text{1},\text{2},\ldots ,n\\ s^{-}\geq \text{0},s^{+}\geq \text{0} \end{array}\right.\;$$ (2)

In the above formula, i = 1, 2, …, n represents the DMUs, and x, y are the input and output vectors, respectively. λ_i_ denotes the weight of the i DMU when the i_0_ DMU is being evaluated for efficiency. e represents the slack vector; εis a non-Archimedean infinitesimal; θ is the efficiency score of the i DMU. The DEA model yields efficiency values in the range of 0–1. s^+^ and s^-^ are the slack variables, representing the differences between the actual and target values of outputs and inputs, respectively. These slack values indicate the direction and extent to which input-output indicators of national fitness public service in various counties and districts can be improved. Eq (3) for the slack variables is:


$$S_{k,i}=x_{k,i}-\sum {\mathrm{\backslash nolimits}}_{k=\text{1}}^{K}\lambda_{i}x_{k,i}$$ (3)

In the above formula for slack variables, k = 1, 2, …, K, and i = 1, 2, …, n. S_k,i_ represents the k input slack for the i DMU

If *θ = 1*, and *S*^*+*^*=S*^*-*^* = 0*, then the DMU is DEA efficient.

If *θ = 1*, but *S*^*+*^*≠0* or *S*^*-*^* ≠ 0*, then the DMU is weakly DEA efficient.

If *θ < 1*, then the DMU is DEA inefficient.

In the second stage, the slack variables derived from the first stage are partitioned into three components: environmental effects, random factors, and managerial inefficiency [49]. During the decomposition of the slack variables, SFA employed to disentangle managerial inefficiency and statistical noise from the composite error term, an additive adjustment is then implemented to place all DMUs under a standardized environmental condition. Accordingly, following Fried et al., adopts the approach of estimating a separate regression for each input slack variable—a linear stochastic frontier functional form with an additive composite error structure (hereafter referred to as the linear stochastic frontier model),helps avoid sample reduction and potential losses in estimation stability, while allowing the environmental component to be netted out from inputs in a linear manner [48]. Due to the input-oriented characteristics of the first stage, only the input slack variables are subject to SFA regression. The SFA regression function Eq (4) is delineated as follows:


$$S_{k,i}=f(Z_{i};\beta_{n})+\nu_{k,i}+\mu_{k,i};i=\text{1},\text{2},\cdots ,I;n=\text{1},\text{2},\cdots ,N$$ (4)

Among them, S_k,i_ represents the k input slack of the i DMU; Z_i_ is the environmental variable, β_n_ is the coefficient of the environmental variable; v_k,i_ + u_k,i_ is the composite error term, where v_k,i_ represents random error, and u_k,i_ represents managerial inefficiency. Here,v~ N(0,σv2) is the random error term, indicating the impact of random disturbances on the input slack variable; μ is managerial inefficiency, indicating the impact of managerial factors on the input slack variable, assumed to follow a **truncated normal distribution at zero**, namely $\mu \sim N^{+}\left(\text{0},\sigma_{\mu }^{\text{2}}\right)$. To achieve the goal of eliminating environmental and random factors, the formula is adjusted so that all DMUs operate under the same environment, the calculation formula Eq (5) is:


$$X_{ni}^{A}=X_{ni}+[max(f(Z_{i};{\overset{\wedge }{\beta }}_{n}))-f(Z_{i};{\overset{\wedge }{\beta }}_{n})]+[max(\nu_{ni})-\nu_{ni}]$$

i=1,2,⋯,I;n=1,2,⋯,N (5)

In this context: ${\text{X}}_{\text{ni}}^{\text{A}}$ represents the adjusted input; ${\text{X}}_{\text{ni}}$ is the unadjusted input; $\left[\text{max}\left(\text{f}\left({\text{z}}_{\text{i}};{\hat{\beta }}_{\text{n}}\right)-\text{f}\left({\text{z}}_{\text{i}};{\hat{\beta }}_{\text{n}}\right)\right)\right]$ denotes the adjustment for external environmental factors; $\left[\text{max}\left({\text{v}}_{\text{ni}}\right)-{\text{v}}_{\text{ni}}\right]$aligns all DMUs (county-level administrative regions) to a common environmental level. This adjustment effectively eliminates the influence of Economic development (per capita GDP), sports and fitness environment (regional urbanization rate), industrial structure (tertiary industry value added/ GDP) and Accessibility constraint of National Fitness public services (population density) on districts and county areas.

**Fig 1:**
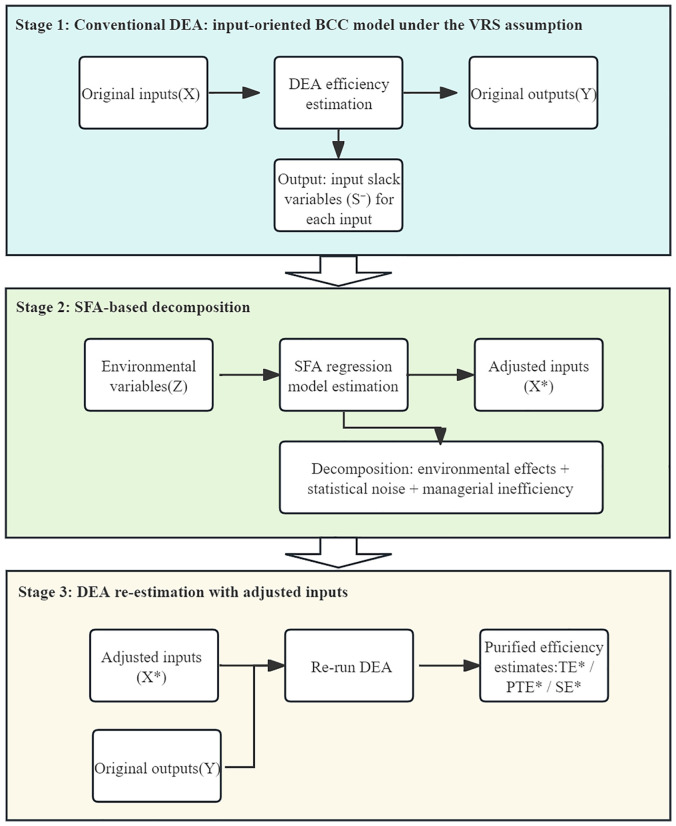
Flowchart of the Three-Stage DEA Procedure.

In the third stage, subsequent to the removal of environmental and stochastic factors in the second stage, eliminating these factors from the input slack variables—adjusted input variables are calculated by combining the original input data with the adjustments. With the modified input variables, the efficiency calculation from the first stage is reiterated to derive the national fitness public service efficiency scores for each county and districts, now devoid of environmental and random factors. These results exhibit greater objectivity than those from the first stage (Fig 1).

## Results

### Descriptive analysis of indicators

As shown in Table 3, the coefficients of variation for fiscal input, the composite facility index, GDP, sports and fitness environment, and accessibility of National Fitness public services all exceeds 0.4, indicates substantial cross-county disparities among Yunnan Province’s 129 county-level administrative divisions in per-capita local general public budget expenditure, GDP per capita, urbanization rate, and the actual provision of sports facilities, suggesting a notable degree of spatial and structural imbalance.

**Table 3:** Units and summary statistics of all variables.

Indicator	Units	M	SD	Min	Max	CV
Per Capita Local General Public Budget Expenditure	CNY per person	10890	6051	3837	47826	0.56
Number of social sports instructors per 1,000 population	persons per 1,000 population	2.74	0.74	1.12	6.39	0.27
Per-capita sports venue area	m² per person	2.57	0.83	0.78	7.61	0.32
Composite facility index	weighted by service-radius categories	1.62	0.81	0.25	4.81	0.50
Proportion of residents who participate in regular physical exercise	%	39.75%	7.61%	17.67%	63.17%	0.19
Pass rate of the National Physical Fitness Measurement Standards	%	92.80%	1.90%	88.08%	98.43%	0.02
Per-capita GDP	CNY per person	58041	26844	19064	185336	0.46
Regional Urbanization Rate	%	44.69%	17.95%	16.42%	99.32%	0.40
tertiary industry value added/ GDP	%	47.08%	10.29%	25.88%	84.11%	0.22
population density	persons/km²	147.51	202.98	6.94	17911	1.37

CV, coefficient of variation; GDP, Gross Domestic Product

### The First stage:the standard DEA model

Uses the DEAP 2.1 software to compute the BCC model with VRS, utilizing an output-oriented approach to optimize output while maintaining fixed inputs. The efficiency throughout 129 districts and counties in Yunnan Province in 2023 is obtained, as presented in Table 4.

**Table 4:** Efficiency of public service for national fitness in municipal districts and county area of 2023 Yunnan province in the first Stage.

Region	Efficiency	Efficiency Range	TE		PTE		SE	
			Quantity	proportion	Quantity	proportion	Quantity	proportion
Municipal district	Effective	θ = 1	3	17.65%	7	41.18%	3	17.65%
		0.8 ≤ θ < 1	7	41.18%	4	23.53%	14	82.35%
	Ineffective	θ < 0.8	7	41.18%	6	35.29%	0	0.00%
	Mean		0.858		0.884		0.972	
County area	Effective	θ = 1	14	12.50%	19	16.96%	14	12.50%
		0.8 ≤ θ < 1	37	33.04%	37	33.04%	98	87.50%
	Ineffective	θ < 0.8	61	54.46%	56	50.00%	0	0.00%
	Mean		0.794		0.816		0.973	

TE, technical efficiency; PTE, pure technical efficiency; SE, Scale efficiency

The TE of public services for National Fitness across Yunnan Province’s 17 districts and 112 county areas from 0.541 to 1, indicating substantial inter-county-level administrative division heterogeneity in efficiency. The mean TE for districts and county areas is 0.858 and 0.794, respectively, implying potential improvements of 14.2% and 20.6%. Moreover, 3 districts and 14 county areas achieve the frontier value of 1, accounting for 17.65% of all districts and 12.50% of all county areas. In addition, 58.83% of districts fall within the relatively efficient interval (0.8 ≤ θ ≤ 1), compared with only 45.54% of county areas. Collectively, relative to county areas, districts exhibit a higher likelihood of reaching the frontier or operating within the efficient range, and are better able to transform inputs into a higher Proportion of residents who participate in regular physical exercise and Pass rate of the National Physical, thereby contributing to improvements in Yunnan’s overall efficiency in National Fitness public service provision.

From efficiency decomposition, the mean SE is 0.972 for districts and 0.973 for county areas. SE is generally high in both groups and exhibits no meaningful differences, indicates that the input scale of National Fitness public services in districts and county areas is relatively well aligned with key outputs—the proportion of residents who regularly participate in physical exercise and the pass rate of the National Physical Fitness Standards —and is close to the optimal scale of production. In practical terms, this suggests that inputs have largely avoided extensive or large-scale idle capacity and waste.

In terms of PT, The average PTE values for districts and county areas were 0.844 and 0.816, respectively. Nonetheless, 35.29% of districts and 50.00% of county regions were categorized as inefficient. In 2023, overall PTE in Yunnan Province was predominantly inferior to scale efficiency. The BCC model, which assesses TE as the product of PTE and SE, indicates that the majority of inefficiencies in Public Services for National Fitness in districts and county areas of Yunnan Province in 2023 were due to comparatively low PTE. Moreover, within county areas, Shangri-La City, Lanping County, Yongren County, and Shiping County demonstrated PTE markedly inferior to the average. Shangri-La City, renowned for its advantageous climate and distinctive cultural traditions, drew more than 31 million tourists in 2024. The principal investments in facilities including public stadiums, fitness centers, and swimming pools were predominantly aimed at sustaining the local tourism industry rather than fostering regular sports participating among inhabitants, has posed challenges when enhancing the effectiveness of Public Services for National Fitness.

**Fig 2:**
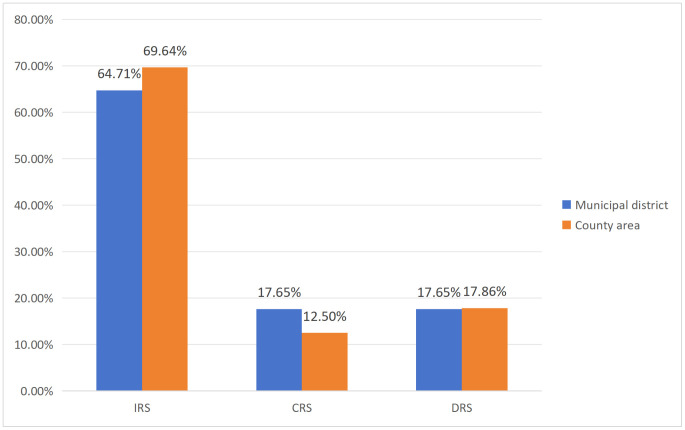
Distribution of Scale Returns of Public Fitness Services inurban districts and Counties of Yunnan. IRS, Increasing Returns to Scale; CRS, Constant Returns to Scale; DRS, Decreasing Returns to Scale.

Fig 2 demonstrates that 11 districts and 78 county areas exhibit increasing returns to scale (IRS), representing 64.71% and 69.64% respectively, indicates that the majority of these regions can enhance their production efficiency frontier by expanding resource inputs such as facilities, human resources, and finances, thereby markedly enhancing the efficiency of public services for national fitness. Conversely, 3 districts and 20 county areas demonstrate decreasing returns to scale (DRS), accounting for 17.65% and 17.86%. In these areas, augmented resource inputs may lead to diminishing returns in the efficiency of sports and fitness public services. Such inefficiencies may obstruct the sustainable advancement of public service systems for sports and fitness, so constraining positive effects on the proportion of individuals engaged in regular physical activities and generic community health. In specific areas of Yunnan Province, the development of sports parks and large-scale venues has become markedly homogeneous; these projects fulfill fundamental requirements for green space ratios and functional designs, primarily emphasizing traditional sports facilities such as football, basketball, and badminton courts. These developments fail to align with the specific needs of local residents or capitalize on distinctive cultural resources, leading to underutilized or capitalize on distinctive. Consequently, this results in an imbalanced relationship between the allocation of facilities, human resources, and finances, and the actual benefits derived from public services, elevates per-unit costs of public service resource consumption, and diminishes the overall efficiency of the sports and fitness public service system.

### The Second Stage: SFA regression

The efficiency results from the first stage neglected the influence of environmental variables on the efficiency. Therefore, it is imperative to utilize the SFA regression outcomes to adjust for these environmental variables, thus ensuring that each district and county area is assessed and compared under equitable conditions, aims to attain a more precise assessment of efficiency values for national fitness across districts and county areas.

As reported in Table 5, SFA results shows σ² is significantly positive for all four categories of input indicators, while γ is generally close to 1. In addition, the likelihood-ratio (LR) tests for the presence of the inefficiency term are all significant at 1%. These findings indicate slack variables for the four input categories contain a substantial systematic inefficiency component. Accordingly, incorporating environmental variables and applying an SFA framework to disentangle environmental effects, statistical noise, and managerial inefficiency constitutes a necessary prerequisite for environmental neutralization and noise correction. Moreover, consistent with the logic of the SFA, the signs of the estimated coefficients should be interpreted from a redundancy–efficiency perspective: larger input slacks imply greater input redundancy at a given output level and thus lower efficiency [49]. A negative coefficient indicates that an increase in the corresponding environmental factor is associated with reduced redundancy and improved efficiency, whereas a positive coefficient implies the opposite.

**Table 5:** SFA Regression of input slack variables (linear stochastic frontier).

Item	Fiscal Investment		Human resources input		Facility input 1		Facility input 2	
	CV	T-values	CV	T-values	CV	T-values	CV	T-values
Constant	−3480***	−13	0.023***	9.500	0.045**	2.370	0.015	1.060
per-capita GDP	0.026***	5.070	−0.001***	−4.530	−0.001	−0.026	0.001	0.168
Urbanization Rate	−6860***	−22.900	0.213**	2.280	0.151***	5.560	0.036	0.679
Industrial structure	8050***	21.700	−0.156***	−14.500	−0.215***	−10.700	−0.066	−1.630
population density	0.614	0.536	−0.001**	−2.170	−0.001***	−2.150	−0.001	−0.578
sigma-squared	37800000***	37700000	0.787***	7.640	0.633***	7.010	0.444***	32.500
gamma	0.996***	307	1***	124000	1***	3080000	1***	11100000
LR	66.3***		61.8***		71.6***		86.4***	

CV, Coefficient value; LR, Likelihood Ratio; *p < 0.1, **p < 0.05, ***p < 0.01.

With respect to the environmental effects on fiscal input slack, the estimated coefficients for GDP per capita and the regional industrial structure are significantly positive, whereas the coefficient for the urbanization rate is significantly negative; the coefficient for population density is not statistically significant. This suggests, on the one hand, that county-level administrative division with higher economic development and a more service-oriented industrial structure tend to exhibit a stronger inclination to meet higher-level public service demands and to enhance urban vitality and image, which is associated with greater redundancy in fiscal inputs for National Fitness public services. On the other hand, population agglomeration and improvements in the spatial structure can raise the marginal productivity of fiscal spending: a higher level of urbanization facilitates the more concentrated allocation of sports facilities and public service programs, thereby spreading fixed costs over a larger service base. As a result, a given scale of fiscal expenditure can serve more users and support higher utilization frequency, leading to a contraction of fiscal slack. By contrast, population density does not exhibit a statistically significant association with fiscal input redundancy.

Regarding the environmental effects on human-resource input slack, the coefficients on GDP per capita, regional industrial structure, and population density are significantly negative, whereas the coefficient on the urbanization rate is significantly positive. In more economically developed, more service-oriented, and more densely populated areas, the allocation of social sports instructors is more compact at a given output level—each instructor supports a larger volume of service output—thereby resulting in lower redundancy. In addition, the positive association between urbanization and human-resource slack suggests that highly urbanized areas may experience a certain degree of misallocation of human resources in the process of meeting increasingly diversified fitness demands, leading to relatively higher redundancy [50].

With respect to the environmental effects on slack in per capita sports venue area, the coefficient on the urbanization rate is significantly positive, whereas the coefficients on regional industrial structure and population density are significantly negative; the coefficient on GDP per capita is not statistically significant. These findings suggest that industrial upgrading and population agglomeration help reduce inefficient underutilization of public sports venues by expanding the user base and enabling stronger coupling with service-sector, market-oriented activities, thereby lowering idle capacity. By contrast, although urbanization tends to promote the concentrated construction of sports facilities, the lagged adjustment of residents’ physical activity habits can generate a structural mismatch between the supplied sports spaces and users’ preferences,thereby resulting in input redundancy which has been documented in multiple countries [51– 52].

For the environmental effects on slack in the composite facility input, which is constructed based on the quantities and service radii of seven categories of sports venues, the estimated coefficients for all four environmental variables do not pass conventional tests of statistical significance. Nevertheless, the estimated γ remains close to 1 and the LR test is statistically significant, indicating that the inefficiency component underlying redundancy in the composite facility input is not driven by these four environmental factors. This pattern is consistent with the policy-oriented and institutionally driven nature of composite facility provision [53– 54], which is shaped less by immediate environmental conditions and more by administrative mandates and institutional arrangements.

To assess the sensitivity of the second-stage estimates to alternative functional-form specifications, Table 6 reports results obtained using both a Cobb–Douglas stochastic frontier and a linear stochastic frontier specification. Overall, the estimated effects of the environmental variables across the four input-slack equations exhibit a high degree of consistency: for the major environmental determinants, the coefficient signs and inferences regarding statistical significance are largely aligned under both functional forms. This indicates that the results regarding the mechanisms through which environmental conditions, including macroeconomic development, urbanization, industrial structure, and service accessibility, affect efficiency do not hinge on any single frontier specification, but instead demonstrate strong robustness to reasonable variations in functional-form assumptions.

**Table 6:** SFA Regression of input slack variables (Cobb-Douglas stochastic frontier).

Item	Fiscal Investment		Human resources input		Facility input 1		Facility input 2	
CV	T-values	CV	T-values	CV	T-values	CV	T-values
Constant	−2900***	−10.200	0.012***	0.215	0.002***	0.194	0.002	0.321
per-capita GDP	0.028***	9.050	−0.001***	−0.148	−0.001	−0.222	0.001	0.249
Urbanization Rate	−4870***	−5.040	0.013***	0.086	0.020***	0.427	0.001	0.095
Industrial structure	5990***	6.730	−0.048***	−0.225	−0.019***	−0.353	−0.008	−0.293
population density	−1.21	−0.781	−0.001***	−0.02	−0.001***	−0.343	−0.001	−0.069
sigma-squared	37800000***	36900000	0.824***	33.700	0.642***	11	0.382***	11.4
gamma	0.994***	264	1***	62300000	1***	564000	1***	114000000
LR	71***		62.4***		72.3***		87.21***	

CV, Coefficient value; LR, Likelihood Ratio; *p < 0.1, **p < 0.05, ***p < 0.01

### The Third Stage:DEA model analysis

In the second stage, environmental variables were excluded by SFA regression. The efficiency values for districts and county areas in the third stage are presented in Table 7. The mean TE for districts and county areas are 0.919 and 0.824, respectively, indicating that there remains a potential for improvement of 8.1% and 17.6%. From the TE perspective, relative to Stage 1, 23.53% of districts improved to the frontier with TE = 1, whereas the share of county areas with TE = 1 declined to 11.61%. In addition, 29.41% of districts and 9.82% of county areas fall within the relatively efficient interval. After netting out environmental variable’s influence, the efficiency gap in National Fitness public service provision between districts and county areas becomes more pronounced, further corroborating the significant effects of environmental conditions on efficiency outcomes.

**Table 7:** Efficiency of public fitness services in municipal districts and county areas of Yunnan province in the third stage in 2023.

Region	Efficiency	Efficiency Range	TE		PTE		SE	
			Quantity	proportion	Quantity	proportion	Quantity	proportion
Municipal district	Effective	θ = 1	7	41.18%	8	47.06%	7	41.18%
		0.8 ≤ θ < 1	8	47.06%	8	47.06%	10	58.82%
	Ineffective	θ < 0.8	2	11.76%	1	5.88%	0	0.00%
	Mean		0.919		0.928		0.989	
County area	Effective	θ = 1	13	11.61%	18	16.07%	15	13.39%
		0.8 ≤ θ < 1	49	43.75%	55	49.11%	97	86.61%
	Ineffective	θ < 0.8	50	44.64%	39	34.82%	0	0.00%
	Mean		0.824		0.844		0.975	

TE, technical efficiency; PTE, pure technical efficiency; SE, Scale efficiency

**Fig 3:**
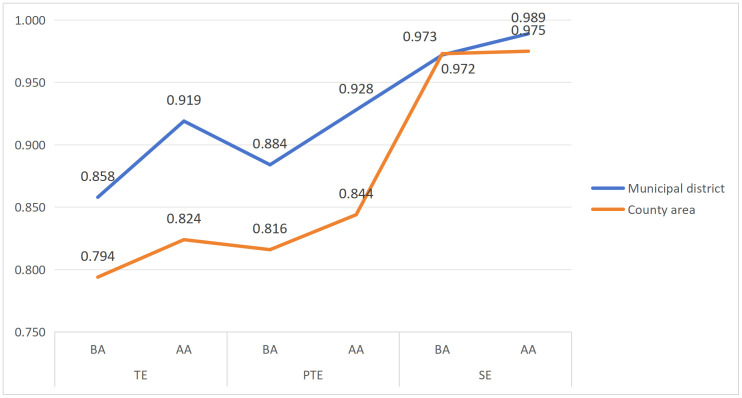
Efficiency of municipal districts and county areas in the first and third stages. BA, Before adjustment;AA, After adjustment;TE, Technical Efficiency;PTE, Pure Technical Efficiency;SE, Scale Efficiency.

As shown in Table 7 and Fig 3, after adjusting for and netting out environmental effects, a comparison of efficiency outcomes between Stages one and three indicates both districts and county areas exhibit clear improvement trends in TE and PTE. Specifically, TE increases by 6.10% for districts and 3.00% for county areas, while PTE rises by 4.40% and 2.80%, respectively. By contrast, SE increases only marginally, by 1.70% for districts and 0.20% for county areas, indicates prior to controlling for environmental factors, the TE, PTE, and SE of National Fitness public services in both districts and county areas were substantially underestimated. After adjustment, SE is generally higher than PTE for both groups, implying that further efficiency gains and system development are constrained primarily by managerial and technical limitations, rather than by scale-related factors.

**Fig 4:**
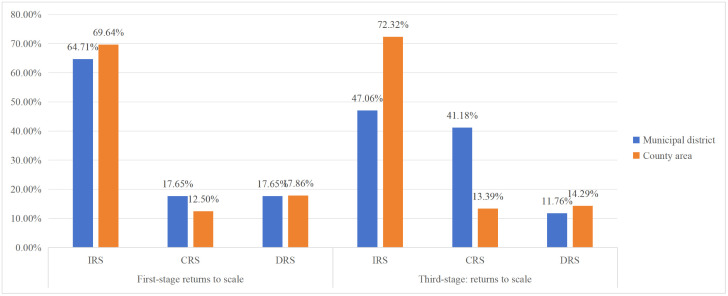
Returns to scale of municipal districts and county areas in the first and third stages. IRS, Increasing Returns to Scale; CRS, Constant Returns to Scale; DRS, Decreasing Returns to Scale.

As illustrated in Fig 4, after adjusting for and eliminating environmental factors, a comparison of the returns to scale Stages one and three indicates that, among districts, 23.53% of those previously operating under IRS and 5.89% of those under DRS are reclassified as operating under CRS. For county areas, the shares operating under IRS and CRS rise to 72.23% and 13.29%, respectively, while the proportion under DRS decreases correspondingly. These results suggest that, environmental conditions exert significant effects on efficiency, and that the post-adjustment efficiency estimates for both districts and county areas provide a more accurate reflection of underlying performance. Moreover, the Stage three returns-to-scale distribution in Fig 4 further indicates that the higher share of CRS among municipal districts, together with the higher share of IRS among county areas, corroborates that the development of National Fitness public services in districts is constrained primarily by limited scope for further scale expansion, whereas county areas remain more constrained by managerial and technical capabilities.

Based on the Stage three efficiency rankings of each county-level administrative division, Spearman rank correlation coefficients were calculated between the BCC and CCR models and between the BCC and SBM models. As reported in Table 8, the rank correlations for BCC versus CCR and BCC versus SBM are 0.82 and 0.87, respectively, indicating a high degree of concordance in the efficiency ordering of county-level administrative divisions/regions across different technological assumptions and measurement approaches. These results suggest that the study’s conclusions are strongly robust to alternative model specifications.

**Table 8:** Spearman Rank Correlation of County-Level administrative division Efficiency Rankings between DEA Models (BCC, CCR, and SBM).

Prefecture-level jurisdiction	county-level district	BCC TE (rank)	CCR TE (rank)	SBM efficiency (rank)
Kunming City	Wuhua District	0.96 (30)	0.99 (19)	1.00 (14)
	Panlong District	0.99 (21)	0.99 (17)	0.78 (39)
	Guandu District	1.00 (6)	1.00 (2)	1.00 (6)
	Xishan District	1.00 (16)	1.00 (8)	1.00 (15)
	Dongchuan District	0.87 (48)	0.96 (44)	0.75 (45)
	Chenggong District	1.00 (2)	1.00 (4)	1.00 (2)
	Jinning District	1.00 (11)	0.99 (14)	1.00 (10)
	Fumin County	1.00 (4)	0.98 (26)	1.00 (4)
	Yiliang County	1.00 (18)	0.97 (29)	1.00 (17)
	Shilin County	0.91 (40)	0.96 (40)	0.83 (33)
	Songming County	0.98 (24)	0.97 (35)	0.79 (37)
	Luquan County	1.00 (12)	1.00 (11)	1.00 (11)
	Xundian County	1.00 (17)	0.95 (54)	1.00 (16)
	Anning City	0.92 (36)	0.96 (46)	0.77 (40)
Zhaotong City	Zhaoyang District	0.79 (83)	0.95 (58)	0.63 (90)
	Ludian County	0.78 (84)	0.94 (83)	0.68 (75)
	Qiaojia County	0.73 (104)	0.93 (103)	0.62 (97)
	Yanjin County	0.98 (25)	0.98 (22)	0.91 (24)
	Daguan County	0.76 (97)	0.95 (57)	0.66 (82)
	Yongshan County	0.75 (100)	0.93 (99)	0.68 (72)
	Suijiang County	0.68 (119)	0.93 (100)	0.63 (92)
	Zhenxiong County	0.78 (86)	0.95 (68)	0.63 (91)
	Yiliang County	0.80 (75)	0.96 (41)	0.71 (60)
	Weixin County	0.78 (87)	0.94 (89)	0.73 (52)
	Shuifu City	0.83 (63)	0.96 (43)	0.65 (84)
Qujing City	Qilin District	0.98 (27)	0.98 (25)	0.71 (61)
	Zhanyi District	0.83 (65)	0.94 (76)	0.65 (85)
	Malong District	0.69 (118)	0.94 (86)	0.61 (102)
	Luliang County	0.84 (61)	0.96 (50)	0.62 (96)
	Shizong County	0.77 (93)	0.94 (85)	0.59 (107)
	Luoping County	0.82 (70)	0.94 (87)	0.66 (81)
	Fuyuan County	0.86 (54)	0.94 (97)	0.73 (51)
	Huize County	0.70 (113)	0.92 (125)	0.59 (108)
	Xuanwei City	0.75 (98)	0.92 (119)	0.54 (115)
Yuxi City	Hongta District	1.00 (8)	0.97 (33)	0.67 (77)
	Jiangchuan District	1.00 (9)	0.97 (31)	1.00 (8)
	Tonghai County	1.00 (15)	0.98 (24)	1.00 (13)
	Huaning County	0.93 (34)	0.95 (67)	0.91 (25)
	Yimen County	0.98 (22)	0.96 (45)	0.94 (22)
	Eshan County	0.75 (101)	0.92 (121)	0.65 (86)
	Xinping County	0.83 (64)	0.94 (91)	0.76 (44)
	Yuanjiang County	0.95 (31)	0.96 (49)	0.92 (23)
	Chengjiang City	0.92 (37)	0.93 (110)	0.87 (27)
Baoshan City	Longyang District	0.87 (51)	0.97 (27)	0.69 (70)
	Shidian County	0.87 (49)	0.93 (98)	0.81 (35)
	Longling County	0.72 (107)	0.93 (111)	0.67 (78)
	Changning County	0.72 (109)	0.92 (114)	0.65 (88)
	Tengchong City	0.91 (41)	1.00 (7)	0.79 (36)
Chuxiong Prefecture	Chuxiong City	0.91 (39)	0.99 (13)	0.74 (49)
	Lufeng County	0.97 (28)	1.00 (12)	0.96 (20)
	Shuangbai County	0.75 (99)	0.94 (93)	0.42 (125)
	Mouding County	0.69 (117)	0.93 (104)	0.61 (101)
	Nanhua County	0.79 (80)	0.92 (116)	0.67 (79)
	Yao’an County	0.79 (78)	0.93 (101)	0.71 (63)
	Dayao County	0.73 (106)	0.91 (126)	0.58 (109)
	Yongren County	0.62 (123)	0.93 (109)	0.38 (128)
	Yuanmou County	0.72 (108)	0.92 (113)	0.61 (99)
	Wuding County	0.71 (110)	0.93 (108)	0.63 (93)
Honghe Prefecture	Gejiu City	0.73 (105)	0.94 (79)	0.56 (113)
	Kaiyuan City	0.67 (120)	0.92 (118)	0.63 (95)
	Mengzi City	0.89 (44)	0.95 (60)	0.69 (68)
	Mile City	0.91 (42)	0.96 (39)	0.81 (34)
	Pingbian County	0.88 (47)	0.96 (42)	0.75 (48)
	Jianshui County	0.87 (50)	0.94 (95)	0.70 (65)
	Shiping County	0.62 (124)	0.93 (107)	0.51 (120)
	Luxi County	0.80 (76)	0.95 (55)	0.65 (87)
	Yuanyang County	1.00 (19)	0.97 (30)	1.00 (18)
	Honghe County	0.94 (33)	0.97 (28)	0.90 (26)
	Jinping County	1.00 (10)	0.97 (37)	1.00 (9)
	Lüchun County	1.00 (13)	0.99 (18)	1.00 (12)
	Hekou County	1.00 (7)	1.00 (3)	1.00 (7)
Wenshan Prefecture	Wenshan City	0.79 (82)	0.94 (90)	0.41 (126)
	Yanshan County	0.92 (38)	1.00 (9)	0.86 (28)
	Xichou County	0.77 (90)	0.95 (73)	0.68 (73)
	Malipo County	0.98 (26)	0.97 (32)	0.86 (29)
	Maguan County	0.82 (71)	0.98 (21)	0.75 (46)
	Qiubei County	0.84 (58)	0.99 (15)	0.72 (56)
	Guangnan County	0.79 (79)	0.99 (16)	0.51 (119)
	Funing County	0.84 (59)	0.98 (23)	0.68 (71)
Pu’er City	Simao District	1.00 (14)	1.00 (6)	0.78 (38)
	Ning’er County	0.70 (115)	0.95 (65)	0.64 (89)
	Mojiang County	0.77 (91)	0.92 (122)	0.73 (53)
	Jingdong County	0.78 (85)	0.92 (117)	0.68 (74)
	Jinggu County	0.86 (53)	0.94 (96)	0.73 (54)
	Zhenyuan County	0.70 (116)	0.93 (112)	0.61 (100)
	Jiangcheng County	0.61 (126)	0.92 (124)	0.55 (114)
	Menglian County	0.66 (121)	0.93 (105)	0.60 (105)
	Lancang County	0.74 (103)	0.94 (74)	0.66 (83)
	Ximeng County	0.77 (92)	0.94 (78)	0.60 (106)
Xishuangbanna Prefecture	Jinghong City	0.83 (67)	0.95 (63)	0.41 (127)
	Menghai County	0.78 (88)	0.94 (88)	0.61 (104)
	Mengla County	0.80 (77)	0.94 (82)	0.71 (58)
Dali Prefecture	Dali City	1.00 (3)	1.00 (5)	1.00 (3)
	Yangbi County	0.60 (128)	0.91 (127)	0.52 (118)
	Xiangyun County	0.84 (62)	0.96 (38)	0.70 (64)
	Binchuan County	1.00 (1)	1.00 (1)	1.00 (1)
	Midu County	0.77 (94)	0.94 (80)	0.69 (69)
	Nanjian County	0.83 (66)	0.95 (69)	0.76 (43)
	Weishan County	0.76 (96)	0.93 (106)	0.62 (98)
	Yongping County	0.76 (95)	0.94 (94)	0.67 (76)
	Yunlong County	1.00 (20)	0.94 (92)	1.00 (19)
	Eryuan County	0.91 (43)	0.95 (53)	0.85 (32)
	Jianchuan County	0.75 (102)	0.95 (72)	0.71 (57)
	Heqing County	0.81 (73)	0.95 (62)	0.69 (67)
Dehong Prefecture	Ruili City	0.84 (60)	0.95 (66)	0.74 (50)
	Mangshi City	0.79 (81)	0.94 (81)	0.57 (110)
	Lianghe County	0.78 (89)	0.94 (84)	0.57 (111)
	Yingjiang County	0.70 (114)	0.91 (128)	0.61 (103)
	Longchuan County	0.89 (45)	0.95 (56)	0.77 (42)
Lijiang City	Gucheng District	0.81 (74)	0.95 (70)	0.47 (123)
	Yulong County	0.62 (125)	0.92 (123)	0.52 (117)
	Yongsheng County	0.86 (55)	0.95 (61)	0.71 (59)
	Huaping County	0.97 (29)	0.96 (47)	0.85 (31)
	Ninglang County	0.93 (35)	0.97 (36)	0.77 (41)
Nujiang Prefecture	Lushui City	0.66 (122)	0.92 (115)	0.49 (122)
	Fugong County	0.88 (46)	0.96 (48)	0.56 (112)
	Gongshan County	1.00 (5)	1.00 (10)	1.00 (5)
	Lanping County	0.60 (127)	0.94 (75)	0.53 (116)
Diqing Prefecture	Shangri-La City	0.54 (129)	0.90 (129)	0.36 (129)
	Deqin County	0.81 (72)	0.94 (77)	0.49 (121)
	Weixi County	0.70 (111)	0.92 (120)	0.42 (124)
Lincang City	Linxiang District	0.85 (56)	0.96 (52)	0.71 (62)
	Fengqing County	0.85 (57)	0.95 (64)	0.70 (66)
	Yun County	0.94 (32)	0.97 (34)	0.85 (30)
	Yongde County	0.98 (23)	0.98 (20)	0.95 (21)
	Zhenkang County	0.87 (52)	0.95 (59)	0.75 (47)
	Shuangjiang County	0.83 (68)	0.95 (71)	0.73 (55)
	Gengma County	0.70 (112)	0.93 (102)	0.63 (94)
	Cangyuan County	0.82 (69)	0.96 (51)	0.67 (80)
Rank consistency with BCC		1	0.82	0.87

TE, Technical Efficiency; BCC, Banker Charnes Cooper model; CCR, Charnes Cooper Rhodes model; SBM, Slack-Based Measure model.

## Discussion

After adjusting for and netting out environmental effects, the difference in mean SE between districts and county areas is less than 0.1%, while the mean PTE is 0.928 for districts and 0.844 for county areas. This indicates that the observed efficiency gap in National Fitness public service provision between districts and county areas is driven primarily by disparities in PTE. Consistent with evidence from Spain [55], the UK [56], Korea [57], the United States [58], and Japan [59], the source of efficiency differentials is closely associated with technical and managerial inefficiency and the institutional environment. This suggests that, relative to municipal districts, Yunnan’s county areas have substantial room for improvement in resource allocation and management, and should prioritize strengthening the allocation capacity of key inputs, including sports facilities, fiscal resources, and human resources, together with more effective development of urban spatial resources and the revitalization of underutilized land stocks for enhance efficiency. Besides, The second-stage SFA results further indicate that improving input output performance does not hinge on simply increasing the aggregate amount of fiscal expenditure, facility supply, or staffing. Rather, the critical leverage lies in optimizing the input structure and governance mechanisms under specific environmental constraints. Districts and county areas should therefore coordinate to advance integrated and balanced development, with particular attention to structural redundancy and behavioral spatial mismatch shaped by economic development, urbanization, and population agglomeration patterns, while continuously expanding service types to respond to residents’ rapidly rising demand for sport and physical activity. Finally, After environmental adjustment, 47.06% of municipal districts and 72.32% of county areas operate under IRS. Compared with most developed countries [60] and China’s more developed eastern regions [38,39], the high prevalence of IRS in Yunnan’s county areas reflects a distinct feature of developing regions and highlights the specificity of ethnic minority areas in western China. This provides a representative Chinese case for understanding how economically less developed yet culturally diverse regions can enhance National Fitness public service efficiency through a dual pathway of scale expansion and governance optimization. In practical terms, as inputs such as fiscal resources, human resources, and facility provision increase steadily across municipal districts and county areas, efficiency is expected to improve by a larger margin. This IRS pattern also implies that both districts and county areas are positioned to leverage scale-expansion effects while coordinating resource input with local contexts and the preservation and transmission of regional and ethnic cultural inheritance.

To address the finding that the efficiency gap between municipal districts and county areas is driven primarily by pronounced differences in PTE, proposes the following policy recommendations. County areas ought to adopt the management practices of urban districts in Yunnan Province and other progressive regions in China to enhance their oversight and management standards system, thereby ensuring the effective functioning of the management system. Efforts should concentrate on establishing standards of national fitness public service in county areas, revising planning standards for public sports facilities in counties and cities, piloting implementation strategies for the accessibility of sports facilities in county areas, delineating the types and formats of open facilities, and ensuring the sustained public accessibility of specific public sports facilities. Furthermore, investigate the formulation of assessment criteria or standards for appraising the accessibility of sports venues and amenities in county-level areas to the public. Assess and rank low-cost or publicly accessible sports venues and amenities, allocating award cash to exemplary venues to encourage their sustained affordability or free access. Second, improve the investment mechanism. Integrate the National Fitness program into the economic and social development plans of county areas, incrementally increasing investment to prioritize the enhancement of basic public sports facilities, particularly in impoverished, remote border, and ethnic minority areas. On the other hand, enhance the effectiveness of sports lottery public welfare fund use by standardizing their management and application in county-level regions. Prohibit the retention, misappropriation, or misuse of these money, guaranteeing that all sports lottery public welfare monies are solely designated for sports-related programs. Enhance oversight and evaluation of the distribution and utilization of sports lottery public welfare funds at the county level to guarantee their true benefit to the public and their crucial contribution to furthering the national policy for promoting fitness for all.

In response to the significant influence of environmental variables on service efficiency, the following suggestions are proposed: First, districts and county areas should leverage existing policies aimed at promoting the development of Public Services for National Fitness. The goal is to establish an integrated urban-rural public service system for sports and fitness that benefits all citizens and aligns with Yunnan’s specific conditions. Efforts should focus on breaking the suction effect from urban districts and developed regions by collaboratively accelerating the revision of local regulations and further enhancing policy guarantees. Second, while steadily increasing inputs in finance, human resources, and sports facilities, a variety of measures should be implemented to enrich the content of Public Services for National Fitness, attract more residents to participate, and increase the proportion of individuals engaging in regular sports activities within each county-level administrative division. It is essential to segment public demand, refine and develop the mass sports market, and organize personalized events tailored to the preferences and needs of different age groups, bringing these activities directly to the public to enhance their daily cultural and sports experiences. Furthermore, the integration of National Fitness with tourism should be explored to enhance the recreational nature of mass sports events. Encourage park and cultural tourism park operators to host leisure fitness activities. Outdoor recreational venues such as parks and wetlands should be fully utilized, combining seasonal natural resources to organize themed activities like “flower viewing,” “spring outings,” “park tours,” or cultural festivals, primarily in the form of low-intensity sports such as hiking. This will deepen the role of sports-tourism integration in advancing National Fitness and encourage people to engage in outdoor exercise. Finally, diversify the types of sports venues and facilities. Fully exploit Yunnan’s abundant outdoor sports resources by strengthening the construction of mountain, self-driving, water, ice and snow, and motor sports campsites, as well as hiking, cycling, and mountain climbing trails. Enhance connectivity between outdoor sports destinations and major transportation routes, and improve supporting facilities such as parking, power supply, water supply, sanitation, communication, signage, and emergency rescue systems. Construct new types of fitness venues and facilities, such as air-supported membrane structure fitness centers, ensuring they meet environmental protection and safety standards.

Given that 72.32% of Yunnan’s county areas operate under IRS, the following recommendation is proposed. First, county areas should develop distinctive sports architecture. Building on ongoing efforts to construct sports parks and to address shortages and gaps in National Fitness facilities, county areas should capitalize on their IRS characteristics by closely integrating the sports culture, traditions, and fitness needs of Yunnan’s 25 ethnic minority groups, including the Yi, Dai, and Miao. This entails further refining county-level plans for National Fitness facility development and embedding ethnic cultural elements and contemporary design features into the architectural design of sports venues. Facility appearance and form should remain coordinated with the overall urban style while clearly highlighting local cultural identity, with the aim of creating new landmark buildings that reflect the distinctive regional character of each county. Second, enhance the construction of facilities for traditional sports projects. During the planning of sports parks and national fitness centers, fully consider traditional sports from various regions and ethnic groups. Integrate distinctive, participatory, and experiential ethnic traditional sports—such as archery, zip-lining, swinging, pole climbing, top spinning, and bamboo pole dancing—into sports parks. Improve the construction of training venues for these traditional sports projects and reasonably plan the layout of sports parks. For example, the Ninger Sports Park in Pu’er City adheres to the principle of “the spirit of the times, ethnic spirit, and local characteristics,” incorporating minority sports traditions and actively promoting sports like top spinning and croquet. This not only plays a significant role in inheriting traditional ethnic and folk cultural sports but also fosters talent development in ethnic and folk cultural sports and advances the overall development of ethnic cultural sports. By enhancing public enthusiasm for sports participation, it further promotes the development of traditional ethnic sports and the dissemination of ethnic culture.

## Conclusion

This study is constrained by the availability and consistency of county-level multi-year statistics,such as changing statistical calibers and discontinuous releases, and by incomplete public disclosure of county-level data at the national level. Consequently, several potentially indicators could not be included, such as numbers of sports social organizations and fitness events, and the analysis is largely based on a single-year cross-section. Future work should aim to construct panel or repeated cross-sectional databases (e.g., 3–10 years with consistent definitions) and combine approaches such as DEA–Malmquist, dynamic DEA, and panel SFA to identify efficiency dynamics. Extending the analysis to a national sample and integrating GIS-based spatial methods would further clarify county-level disparities and spatial patterns.

Using three-stage DEA for 129 county-level administrative divisions in Yunnan Province in 2023, including 17 municipal districts and 112 county areas, the results reveal substantial efficiency heterogeneity. In Stage one, TE ranges from 0.541 to 1.000, and the mean TE is 0.858 for municipal districts versus 0.794 for county area, indicating remaining improvement potentials of 14.2% and 20.6%, respectively. The decomposition further shows that SE is generally close to 1 for both groups, with only negligible differences, implying that the observed gap is driven primarily by PTE, namely managerial and technical capability, rather than by scale factors. The Stage two SFA confirms that macro-environmental conditions such as economic development, urbanization, industrial structure, and population agglomeration significantly shape input slacks and the measured efficiency, which supports the necessity of environmental purification and noise correction. After removing environmental effects and statistical noise, Stage three efficiencies rise to 0.919 for municipal districts and 0.824 for county areas, while the PTE gap remains substantial, with values of 0.928 for municipal districts and 0.844 for county areas. This indicates that the urban–county efficiency disparity is dominated by governance and managerial or technical performance rather than by scale. Returns-to-scale patterns show that, after adjustment, a larger share of county areas operate under IRS (72%), suggesting further room for efficiency gains through appropriate expansion combined with improved organization and governance; by contrast, municipal districts converge more toward CRS, implying more limited marginal benefits from simple expansion. Finally, rank consistency across alternative DEA specifications is relatively high, with Spearman correlations of 0.82 and 0.87, indicating that the main findings are robust to model choice. From a policy perspective, improving county-level national fitness public service efficiency in Yunnan should prioritize three coordinated directions. First, governance capacity should be strengthened through clearer standards, monitoring, and performance-oriented management to reduce input redundancy. Second, the composition of fiscal, human, and facility inputs should be optimized under given constraints, with greater emphasis on matching supply with demand and revitalizing existing space resources. Third, urban–rural integration and cross-regional coordination should be advanced, alongside the development of service packages better aligned with local needs and ethnic-cultural contexts, thereby leveraging increasing-returns scale gains while upgrading governance capacity.

## Supporting information

S1 Table Units and summary statistics of all variables. CV, coefficient of variation; GDP, Gross Domestic Product.
(DOC)

S2 Table Efficiency of public service for national fitness in municipal districts and county area of 2023 Yunnan province in the first Stage. TE, technical efficiency; PTE, pure technical efficiency; SE, Scale efficiency.
(DOC)

S3 Table SFA Regression of input slack variables (linear stochastic frontier). CV, Coefficient value; LR, Likelihood Ratio;*p < 0.1, **p < 0.05, ***p < 0.01.
(DOC)

S4 Table SFA Regression of input slack variables (Cobb-Douglas stochastic frontier). CV, Coefficient value; LR, Likelihood Ratio;*p < 0.1, **p < 0.05, ***p < 0.01.
(DOC)

S5 Table Efficiency of public fitness services in municipal districts and county areas of Yunnan province in the third stage in 2023. TE, technical efficiency; PTE, pure technical efficiency; SE, Scale efficiency.
(DOC)

S6 Table Spearman Rank Correlation of County-Level administrative division Efficiency Rankings between DEA Models (BCC, CCR, and SBM). TE, Technical Efficiency; BCC, Banker Charnes Cooper model; CCR, Charnes Cooper Rhodes model; SBM, Slack-Based Measure model.
(DOC)
