# Supplementary material for: Efficiency evaluation of Chinese Yunnan Province County Area Public Service for sports and fitness based on three-stage DEA model
Source: PLoS One. 2026 Feb 2;21(2):e0340803. doi: 10.1371/journal.pone.0340803 (PMC12863572; doi:10.1371/journal.pone.0340803)
Supplement: S1 Table — CV, coefficient of variation; GDP, Gross Domestic Product. (DOC) [file pone.0340803.s001.doc]

**Table 3. Units and summary statistics of all variables.**

| Indicator | Units | M | SD | Min | Max | CV |
| --- | --- | --- | --- | --- | --- | --- |
| Per Capita Local General Public Budget Expenditure | CNY per person | 10890 | 6051 | 3837 | 47826 | 0.56 |
| Number of social sports instructors per 1,000 population | persons per 1,000 population | 2.74 | 0.74 | 1.12 | 6.39 | 0.27 |
| Per-capita sports venue area | m² per person | 2.57 | 0.83 | 0.78 | 7.61 | 0.32 |
| Composite facility index | weighted by service-radius categories | 1.62 | 0.81 | 0.25 | 4.81 | 0.50 |
| Proportion of residents who participate in regular physical exercise | % | 39.75% | 7.61% | 17.67% | 63.17% | 0.19 |
| Pass rate of the National Physical Fitness Measurement Standards | % | 92.80% | 1.90% | 88.08% | 98.43% | 0.02 |
| Per-capita GDP | CNY per person | 58041 | 26844 | 19064 | 185336 | 0.46 |
| Regional Urbanization Rate | % | 44.69% | 17.95% | 16.42% | 99.32% | 0.40 |
| tertiary industry value added / GDP | % | 47.08% | 10.29% | 25.88% | 84.11% | 0.22 |
| population density | persons/km² | 147.51 | 202.98 | 6.94 | 17911 | 1.37 |

CV, coefficient of variation; GDP, Gross Domestic Product.
