# Supplementary material for: Efficiency evaluation of Chinese Yunnan Province County Area Public Service for sports and fitness based on three-stage DEA model
Source: PLoS One. 2026 Feb 2;21(2):e0340803. doi: 10.1371/journal.pone.0340803 (PMC12863572; doi:10.1371/journal.pone.0340803)
Supplement: S2 Table — TE, technical efficiency; PTE, pure technical efficiency; SE, Scale efficiency. (DOC) [file pone.0340803.s002.doc]

**Table 4. Efficiency of public service for national fitness in municipal districts and county area of 2023 Yunnan province in the first Stage.**

| Region | Efficiency | Efficiency Range | TE | | PTE | | SE | |
| --- | --- | --- | --- | --- | --- | --- | --- | --- |
|  |  |  | Quantity | proportion | Quantity | proportion | Quantity | proportion |
| Municipal district | Effective | θ = 1 | 3 | 17.65% | 7 | 41.18% | 3 | 17.65% |
|  |  | 0.8≤ θ ＜1 | 7 | 41.18% | 4 | 23.53% | 14 | 82.35% |
|  | Ineffective | θ＜ 0.8 | 7 | 41.18% | 6 | 35.29% | 0 | 0.00% |
|  | Mean | | 0.858 | | 0.884 | | 0.972 | |
| County area | Effective | θ= 1 | 14 | 12.50% | 19 | 16.96% | 14 | 12.50% |
|  |  | 0.8≤ θ ＜1 | 37 | 33.04% | 37 | 33.04% | 98 | 87.50% |
|  | Ineffective | θ ＜0.8 | 61 | 54.46% | 56 | 50.00% | 0 | 0.00% |
|  | Mean | | 0.794 | | 0.816 | | 0.973 | |

TE, technical efficiency; PTE, pure technical efficiency; SE, Scale efficiency.
