# Supplementary material for: Efficiency evaluation of Chinese Yunnan Province County Area Public Service for sports and fitness based on three-stage DEA model
Source: PLoS One. 2026 Feb 2;21(2):e0340803. doi: 10.1371/journal.pone.0340803 (PMC12863572; doi:10.1371/journal.pone.0340803)
Supplement: S3 Table — CV, Coefficient value; LR, Likelihood Ratio;*p < 0.1, **p < 0.05, ***p < 0.01. (DOC) [file pone.0340803.s003.doc]

**Table 5. SFA Regression of input slack variables (linear stochastic frontier)**

| Item | Fiscal Investment | | Human resources input | | Facility input 1 | | Facility input 2 | |
| --- | --- | --- | --- | --- | --- | --- | --- | --- |
|  | CV | T-values | CV | T-values | CV | T-values | CV | T-values |
| Constant | -3480*** | -13 | 0.023*** | 9.500 | 0.045** | 2.370 | 0.015 | 1.060 |
| per-capita GDP | 0.026*** | 5.070 | -0.001*** | -4.530 | -0.001 | -0.026 | 0.001 | 0.168 |
| Urbanization Rate | -6860*** | -22.900 | 0.213** | 2.280 | 0.151*** | 5.560 | 0.036 | 0.679 |
| Industrial structure | 8050*** | 21.700 | -0.156*** | -14.500 | -0.215*** | -10.700 | -0.066 | -1.630 |
| population density | 0.614 | 0.536 | -0.001** | -2.170 | -0.001*** | -2.150 | -0.001 | -0.578 |
| sigma-squared | 37800000*** | 37700000 | 0.787*** | 7.640 | 0.633*** | 7.010 | 0.444*** | 32.500 |
| gamma | 0.996*** | 307 | 1*** | 124000 | 1*** | 3080000 | 1*** | 11100000 |
| LR | 66.3*** | | 61.8*** | | 71.6*** | | 86.4*** | |

CV, Coefficient value; LR, Likelihood Ratio;*p < 0.1, **p < 0.05, ***p < 0.01.
