# Supplementary material for: Efficiency evaluation of Chinese Yunnan Province County Area Public Service for sports and fitness based on three-stage DEA model
Source: PLoS One. 2026 Feb 2;21(2):e0340803. doi: 10.1371/journal.pone.0340803 (PMC12863572; doi:10.1371/journal.pone.0340803)
Supplement: S4 Table — CV, Coefficient value; LR, Likelihood Ratio;*p < 0.1, **p < 0.05, ***p < 0.01. (DOC) [file pone.0340803.s004.doc]

**Table 6. SFA Regression of input slack variables (Cobb-Douglas stochastic frontier)**

| Item | Fiscal Investment | | Human resources input | | Facility input 1 | | Facility input 2 | |
| --- | --- | --- | --- | --- | --- | --- | --- | --- |
|  | CV | T-values | CV | T-values | CV | T-values | CV | T-values |
| Constant | -2900*** | -10.200 | 0.012*** | 0.215 | 0.002*** | 0.194 | 0.002 | 0.321 |
| per-capita GDP | 0.028*** | 9.050 | -0.001*** | -0.148 | -0.001 | -0.222 | 0.001 | 0.249 |
| Urbanization Rate | -4870*** | -5.040 | 0.013*** | 0.086 | 0.020*** | 0.427 | 0.001 | 0.095 |
| Industrial structure | 5990*** | 6.730 | -0.048*** | -0.225 | -0.019*** | -0.353 | -0.008 | -0.293 |
| population density | -1.21 | -0.781 | -0.001*** | -0.02 | -0.001*** | -0.343 | -0.001 | -0.069 |
| sigma-squared | 37800000*** | 36900000 | 0.824*** | 33.700 | 0.642*** | 11 | 0.382*** | 11.4 |
| gamma | 0.994*** | 264 | 1*** | 62300000 | 1*** | 564000 | 1*** | 114000000 |
| LR | 71*** | | 62.4*** | | 72.3*** | | 87.21*** | |

CV, Coefficient value; LR, Likelihood Ratio;*p < 0.1, **p < 0.05, ***p < 0.01.
