# Supplementary material for: Efficiency evaluation of Chinese Yunnan Province County Area Public Service for sports and fitness based on three-stage DEA model
Source: PLoS One. 2026 Feb 2;21(2):e0340803. doi: 10.1371/journal.pone.0340803 (PMC12863572; doi:10.1371/journal.pone.0340803)
Supplement: S5 Table — TE, technical efficiency; PTE, pure technical efficiency; SE, Scale efficiency. (DOC) [file pone.0340803.s005.doc]

**Table 7. Efficiency of public fitness services in municipal districts and county areas of Yunnan province in the third stage in 2023**

| Region | Efficiency | Efficiency Range | TE | | PTE | | SE | |
| --- | --- | --- | --- | --- | --- | --- | --- | --- |
|  |  |  | Quantity | proportion | Quantity | proportion | Quantity | proportion |
| Municipal district | Effective | θ = 1 | 7 | 41.18% | 8 | 47.06% | 7 | 41.18% |
|  |  | 0.8≤ θ ＜1 | 8 | 47.06% | 8 | 47.06% | 10 | 58.82% |
|  | Ineffective | θ＜ 0.8 | 2 | 11.76% | 1 | 5.88% | 0 | 0.00% |
|  | Mean | | 0.919 | | 0.928 | | 0.989 | |
| County area | Effective | θ= 1 | 13 | 11.61% | 18 | 16.07% | 15 | 13.39% |
|  |  | 0.8≤ θ ＜1 | 49 | 43.75% | 55 | 49.11% | 97 | 86.61% |
|  | Ineffective | θ ＜0.8 | 50 | 44.64% | 39 | 34.82% | 0 | 0.00% |
|  | Mean | | 0.824 | | 0.844 | | 0.975 | |

TE, technical efficiency; PTE, pure technical efficiency; SE, Scale efficiency.
