# Supplementary material for: Efficiency evaluation of Chinese Yunnan Province County Area Public Service for sports and fitness based on three-stage DEA model
Source: PLoS One. 2026 Feb 2;21(2):e0340803. doi: 10.1371/journal.pone.0340803 (PMC12863572; doi:10.1371/journal.pone.0340803)
Supplement: S6 Table — TE, Technical Efficiency; BCC, Banker Charnes Cooper model; CCR, Charnes Cooper Rhodes model; SBM, Slack-Based Measure model. (DOC) [file pone.0340803.s006.doc]

**Table 8. Spearman Rank Correlation of County-Level administrative division Efficiency Rankings between DEA Models (BCC, CCR, and SBM)**

| **Prefecture-level jurisdiction** | **county-level district** | **BCC TE**  **(rank)** | **CCR TE**  **(rank)** | **SBM efficiency (rank)** |
| --- | --- | --- | --- | --- |
| Kunming City | Wuhua District | 0.96 (30) | 0.99 (19) | 1.00 (14) |
|  | Panlong District | 0.99 (21) | 0.99 (17) | 0.78 (39) |
|  | Guandu District | 1.00 (6) | 1.00 (2) | 1.00 (6) |
|  | Xishan District | 1.00 (16) | 1.00 (8) | 1.00 (15) |
|  | Dongchuan District | 0.87 (48) | 0.96 (44) | 0.75 (45) |
|  | Chenggong District | 1.00 (2) | 1.00 (4) | 1.00 (2) |
|  | Jinning District | 1.00 (11) | 0.99 (14) | 1.00 (10) |
|  | Fumin County | 1.00 (4) | 0.98 (26) | 1.00 (4) |
|  | Yiliang County | 1.00 (18) | 0.97 (29) | 1.00 (17) |
|  | Shilin County | 0.91 (40) | 0.96 (40) | 0.83 (33) |
|  | Songming County | 0.98 (24) | 0.97 (35) | 0.79 (37) |
|  | Luquan County | 1.00 (12) | 1.00 (11) | 1.00 (11) |
|  | Xundian County | 1.00 (17) | 0.95 (54) | 1.00 (16) |
|  | Anning City | 0.92 (36) | 0.96 (46) | 0.77 (40) |
| Zhaotong City | Zhaoyang District | 0.79 (83) | 0.95 (58) | 0.63 (90) |
|  | Ludian County | 0.78 (84) | 0.94 (83) | 0.68 (75) |
|  | Qiaojia County | 0.73 (104) | 0.93 (103) | 0.62 (97) |
|  | Yanjin County | 0.98 (25) | 0.98 (22) | 0.91 (24) |
|  | Daguan County | 0.76 (97) | 0.95 (57) | 0.66 (82) |
|  | Yongshan County | 0.75 (100) | 0.93 (99) | 0.68 (72) |
|  | Suijiang County | 0.68 (119) | 0.93 (100) | 0.63 (92) |
|  | Zhenxiong County | 0.78 (86) | 0.95 (68) | 0.63 (91) |
|  | Yiliang County | 0.80 (75) | 0.96 (41) | 0.71 (60) |
|  | Weixin County | 0.78 (87) | 0.94 (89) | 0.73 (52) |
|  | Shuifu City | 0.83 (63) | 0.96 (43) | 0.65 (84) |
| Qujing City | Qilin District | 0.98 (27) | 0.98 (25) | 0.71 (61) |
|  | Zhanyi District | 0.83 (65) | 0.94 (76) | 0.65 (85) |
|  | Malong District | 0.69 (118) | 0.94 (86) | 0.61 (102) |
|  | Luliang County | 0.84 (61) | 0.96 (50) | 0.62 (96) |
|  | Shizong County | 0.77 (93) | 0.94 (85) | 0.59 (107) |
|  | Luoping County | 0.82 (70) | 0.94 (87) | 0.66 (81) |
|  | Fuyuan County | 0.86 (54) | 0.94 (97) | 0.73 (51) |
|  | Huize County | 0.70 (113) | 0.92 (125) | 0.59 (108) |
|  | Xuanwei City | 0.75 (98) | 0.92 (119) | 0.54 (115) |
| Yuxi City | Hongta District | 1.00 (8) | 0.97 (33) | 0.67 (77) |
|  | Jiangchuan District | 1.00 (9) | 0.97 (31) | 1.00 (8) |
|  | Tonghai County | 1.00 (15) | 0.98 (24) | 1.00 (13) |
|  | Huaning County | 0.93 (34) | 0.95 (67) | 0.91 (25) |
|  | Yimen County | 0.98 (22) | 0.96 (45) | 0.94 (22) |
|  | Eshan County | 0.75 (101) | 0.92 (121) | 0.65 (86) |
|  | Xinping County | 0.83 (64) | 0.94 (91) | 0.76 (44) |
|  | Yuanjiang County | 0.95 (31) | 0.96 (49) | 0.92 (23) |
|  | Chengjiang City | 0.92 (37) | 0.93 (110) | 0.87 (27) |
| Baoshan City | Longyang District | 0.87 (51) | 0.97 (27) | 0.69 (70) |
|  | Shidian County | 0.87 (49) | 0.93 (98) | 0.81 (35) |
|  | Longling County | 0.72 (107) | 0.93 (111) | 0.67 (78) |
|  | Changning County | 0.72 (109) | 0.92 (114) | 0.65 (88) |
|  | Tengchong City | 0.91 (41) | 1.00 (7) | 0.79 (36) |
| Chuxiong Prefecture | Chuxiong City | 0.91 (39) | 0.99 (13) | 0.74 (49) |
|  | Lufeng County | 0.97 (28) | 1.00 (12) | 0.96 (20) |
|  | Shuangbai County | 0.75 (99) | 0.94 (93) | 0.42 (125) |
|  | Mouding County | 0.69 (117) | 0.93 (104) | 0.61 (101) |
|  | Nanhua County | 0.79 (80) | 0.92 (116) | 0.67 (79) |
|  | Yao’an County | 0.79 (78) | 0.93 (101) | 0.71 (63) |
|  | Dayao County | 0.73 (106) | 0.91 (126) | 0.58 (109) |
|  | Yongren County | 0.62 (123) | 0.93 (109) | 0.38 (128) |
|  | Yuanmou County | 0.72 (108) | 0.92 (113) | 0.61 (99) |
|  | Wuding County | 0.71 (110) | 0.93 (108) | 0.63 (93) |
| Honghe Prefecture | Gejiu City | 0.73 (105) | 0.94 (79) | 0.56 (113) |
|  | Kaiyuan City | 0.67 (120) | 0.92 (118) | 0.63 (95) |
|  | Mengzi City | 0.89 (44) | 0.95 (60) | 0.69 (68) |
|  | Mile City | 0.91 (42) | 0.96 (39) | 0.81 (34) |
|  | Pingbian County | 0.88 (47) | 0.96 (42) | 0.75 (48) |
|  | Jianshui County | 0.87 (50) | 0.94 (95) | 0.70 (65) |
|  | Shiping County | 0.62 (124) | 0.93 (107) | 0.51 (120) |
|  | Luxi County | 0.80 (76) | 0.95 (55) | 0.65 (87) |
|  | Yuanyang County | 1.00 (19) | 0.97 (30) | 1.00 (18) |
|  | Honghe County | 0.94 (33) | 0.97 (28) | 0.90 (26) |
|  | Jinping County | 1.00 (10) | 0.97 (37) | 1.00 (9) |
|  | Lüchun County | 1.00 (13) | 0.99 (18) | 1.00 (12) |
|  | Hekou County | 1.00 (7) | 1.00 (3) | 1.00 (7) |
| Wenshan Prefecture | Wenshan City | 0.79 (82) | 0.94 (90) | 0.41 (126) |
|  | Yanshan County | 0.92 (38) | 1.00 (9) | 0.86 (28) |
|  | Xichou County | 0.77 (90) | 0.95 (73) | 0.68 (73) |
|  | Malipo County | 0.98 (26) | 0.97 (32) | 0.86 (29) |
|  | Maguan County | 0.82 (71) | 0.98 (21) | 0.75 (46) |
|  | Qiubei County | 0.84 (58) | 0.99 (15) | 0.72 (56) |
|  | Guangnan County | 0.79 (79) | 0.99 (16) | 0.51 (119) |
|  | Funing County | 0.84 (59) | 0.98 (23) | 0.68 (71) |
| Pu’er City | Simao District | 1.00 (14) | 1.00 (6) | 0.78 (38) |
|  | Ning’er County | 0.70 (115) | 0.95 (65) | 0.64 (89) |
|  | Mojiang County | 0.77 (91) | 0.92 (122) | 0.73 (53) |
|  | Jingdong County | 0.78 (85) | 0.92 (117) | 0.68 (74) |
|  | Jinggu County | 0.86 (53) | 0.94 (96) | 0.73 (54) |
|  | Zhenyuan County | 0.70 (116) | 0.93 (112) | 0.61 (100) |
|  | Jiangcheng County | 0.61 (126) | 0.92 (124) | 0.55 (114) |
|  | Menglian County | 0.66 (121) | 0.93 (105) | 0.60 (105) |
|  | Lancang County | 0.74 (103) | 0.94 (74) | 0.66 (83) |
|  | Ximeng County | 0.77 (92) | 0.94 (78) | 0.60 (106) |
| Xishuangbanna Prefecture | Jinghong City | 0.83 (67) | 0.95 (63) | 0.41 (127) |
|  | Menghai County | 0.78 (88) | 0.94 (88) | 0.61 (104) |
|  | Mengla County | 0.80 (77) | 0.94 (82) | 0.71 (58) |
| Dali Prefecture | Dali City | 1.00 (3) | 1.00 (5) | 1.00 (3) |
|  | Yangbi County | 0.60 (128) | 0.91 (127) | 0.52 (118) |
|  | Xiangyun County | 0.84 (62) | 0.96 (38) | 0.70 (64) |
|  | Binchuan County | 1.00 (1) | 1.00 (1) | 1.00 (1) |
|  | Midu County | 0.77 (94) | 0.94 (80) | 0.69 (69) |
|  | Nanjian County | 0.83 (66) | 0.95 (69) | 0.76 (43) |
|  | Weishan County | 0.76 (96) | 0.93 (106) | 0.62 (98) |
|  | Yongping County | 0.76 (95) | 0.94 (94) | 0.67 (76) |
|  | Yunlong County | 1.00 (20) | 0.94 (92) | 1.00 (19) |
|  | Eryuan County | 0.91 (43) | 0.95 (53) | 0.85 (32) |
|  | Jianchuan County | 0.75 (102) | 0.95 (72) | 0.71 (57) |
|  | Heqing County | 0.81 (73) | 0.95 (62) | 0.69 (67) |
| Dehong Prefecture | Ruili City | 0.84 (60) | 0.95 (66) | 0.74 (50) |
|  | Mangshi City | 0.79 (81) | 0.94 (81) | 0.57 (110) |
|  | Lianghe County | 0.78 (89) | 0.94 (84) | 0.57 (111) |
|  | Yingjiang County | 0.70 (114) | 0.91 (128) | 0.61 (103) |
|  | Longchuan County | 0.89 (45) | 0.95 (56) | 0.77 (42) |
| Lijiang City | Gucheng District | 0.81 (74) | 0.95 (70) | 0.47 (123) |
|  | Yulong County | 0.62 (125) | 0.92 (123) | 0.52 (117) |
|  | Yongsheng County | 0.86 (55) | 0.95 (61) | 0.71 (59) |
|  | Huaping County | 0.97 (29) | 0.96 (47) | 0.85 (31) |
|  | Ninglang County | 0.93 (35) | 0.97 (36) | 0.77 (41) |
| Nujiang Prefecture | Lushui City | 0.66 (122) | 0.92 (115) | 0.49 (122) |
|  | Fugong County | 0.88 (46) | 0.96 (48) | 0.56 (112) |
|  | Gongshan County | 1.00 (5) | 1.00 (10) | 1.00 (5) |
|  | Lanping County | 0.60 (127) | 0.94 (75) | 0.53 (116) |
| Diqing Prefecture | Shangri-La City | 0.54 (129) | 0.90 (129) | 0.36 (129) |
|  | Deqin County | 0.81 (72) | 0.94 (77) | 0.49 (121) |
|  | Weixi County | 0.70 (111) | 0.92 (120) | 0.42 (124) |
| Lincang City | Linxiang District | 0.85 (56) | 0.96 (52) | 0.71 (62) |
|  | Fengqing County | 0.85 (57) | 0.95 (64) | 0.70 (66) |
|  | Yun County | 0.94 (32) | 0.97 (34) | 0.85 (30) |
|  | Yongde County | 0.98 (23) | 0.98 (20) | 0.95 (21) |
|  | Zhenkang County | 0.87 (52) | 0.95 (59) | 0.75 (47) |
|  | Shuangjiang County | 0.83 (68) | 0.95 (71) | 0.73 (55) |
|  | Gengma County | 0.70 (112) | 0.93 (102) | 0.63 (94) |
|  | Cangyuan County | 0.82 (69) | 0.96 (51) | 0.67 (80) |
| Rank consistency with BCC | | 1 | 0.82 | 0.87 |

TE, Technical Efficiency; BCC, Banker Charnes Cooper model; CCR, Charnes Cooper Rhodes model; SBM, Slack-Based Measure model.
